# Association of physical activity and physical performance with tibial cartilage volume and bone area in young adults

**DOI:** 10.1186/s13075-015-0813-0

**Published:** 2015-10-26

**Authors:** Benny Antony, Alison Venn, Flavia Cicuttini, Lyn March, Leigh Blizzard, Terence Dwyer, Marita Cross, Graeme Jones, Changhai Ding

**Affiliations:** Menzies Institute for Medical Research, University of Tasmania, Hobart, Australia; Department of Epidemiology and Preventive Medicine, Monash University, Melbourne, Australia; Institute of Bone and Joint Research, University of Sydney, Sydney, Australia; Murdoch Childrens Research Institute, Melbourne, Australia

## Abstract

**Introduction:**

Physical activity has been recommended to patients with knee osteoarthritis for improving their symptoms. However, it is still controversial if physical activity has effects on joint structures including cartilage volume. The aim of this study was to describe the associations between physical activity and performance measured 5 years prior and tibial cartilage volume and bone area in young adults.

**Methods:**

Subjects broadly representative of the Australian population (*n* = 328, aged 31–41 years, female 47.3 %) were selected from the Childhood Determinants of Adult Health study. They underwent T1-weighted fat-suppressed magnetic resonance imaging (MRI) scans of their knees. Tibial bone area and cartilage volume were measured from MRI. Physical activity (measured using long international physical activity questionnaire (IPAQ)) and performance measures (long jump, leg muscle strength, physical work capacity (PWC_170_)) were measured 5 years prior.

**Results:**

In multivariable analyses, total physical activity (min/week) (β: 0.30 mm^3^, 95 % CI: 0.13,0.47), vigorous (β: 0.54 mm^3^, 95 % CI: 0.13,0.94), moderate (β: 0.34 mm^3^, 95 % CI: 0.01,0.67), walking (β: 0.40 mm^3^, 95 % CI: 0.07,0.72) and IPAQ category (β: 182.9 mm^3^, 95 % CI: 51.8,314.0) were positively associated with total tibial cartilage volume but not tibial bone area. PWC_170_, long jump and leg muscle strength were positively and significantly associated with both total tibial cartilage volume and total tibial bone area; and the associations with tibial cartilage volume decreased in magnitude but remained significant for PWC_170_ and long jump after further adjustment for tibial bone area.

**Conclusion:**

While tibial bone area is affected only by physical performance, total tibial cartilage volume can be influenced by both physical activity and performance in younger adults. The clinical significance suggests a beneficial effect for cartilage but the bone area association was restricted to performance suggesting other factors rather than physical activity may be important.

## Introduction

Knee osteoarthritis (OA) is characterised by the abnormalities of the whole joint structure, including cartilage loss and subchondral bone changes, and clinical symptoms. Magnetic resonance imaging (MRI) has revolutionized research in knee OA because of the clear visualization of structures in the whole knee joint and the ability to manipulate the MR images to generate accurate data such as knee cartilage volume and tibial bone area [1]. Tibial cartilage volume measures the quantity of the tibial cartilage covering the surface of tibial bone and tibial bone area is the cross-sectional surface area of the tibial plateau. Knee cartilage volume is smaller in females than males [2] and in medial tibiofemoral compartment [3] than lateral tibiofemoral compartment, which may explain why knee OA is more common in female patients [4] and in the medial tibiofemoral compartment [5]. Therefore, small knee cartilage volume can be a risk factor for knee OA [6]. Similarly, bone size in a normal young adult is the result of genetics and adaptations to physical stimuli during skeletal growth [7], and a larger tibial plateau in younger adults may be protective by reducing stresses in the joints [8], though tibial bone area has been associated with osteoarthritic changes in middle-aged and older adults [9].

Physical activity has been recommended to patients with knee and hip OA for improving their symptoms [10]. It is still controversial whether physical activity has effects on joint structures. We reported that physical activity is beneficially associated with cartilage volume in healthy children [11, 12]. A systematic review has shown that various physical activities may have different effects on different knee structures [13], and there is limited evidence that there is a positive relationship between physical activity and knee cartilage volume [13]. Most of the studies included in this review were in older adults, with only a few being performed in younger adults. It is possible that the effects of physical activity on knee cartilage volume in younger adults are different from those in older adults. Physical activity increases cortical bone size in the tibia [14], which may influence the tibial surface area due to adaptive mechanisms during growth.

While physical activity reflects behavior, physical performance is operationalized as several measurable health-related phenotypes including cardiorespiratory fitness and muscle performance, which have both genetic and environmental components. Physical performance measures (PPMs) could be a proxy measure of physical activity but there is only moderate correlation between physical activity and PPMs, and physical fitness is a better predictor of cardiovascular risk than physical activity [15]. Some studies focusing on PPMs, such as muscle strength and cardiorespiratory fitness, have shown a protective effect on the structure of the knee [16–18]. It is unknown whether use of PPMs reflects different effects of physical activity on the structure of the knee, particularly in young adults. Therefore, the aims of this study were to describe the associations between physical activity and physical performance measured 5 years prior, and tibial cartilage volume and bone area in young adults.

## Materials and methods

### Subjects

The Childhood Determinants of Adult Health Knee study (CDAH Knee Cartilage study) was conducted during 2008–2010. It was a follow-up study on a subsample (n = 328, mean age 35, range 31–42 years) of participants in the CDAH study (n = 2,410, mean age 31 years) which was conducted during the period of 2004–2006. The CDAH study included Australia-wide subjects ages 26–31 years. The CDAH participants (n = 764) residing in metropolitan Melbourne and Sydney were contacted by mail and invited to participate in the CDAH Knee Cartilage study. Subjects who agreed to participate (n = 529, response rate 69 %) were assessed for their eligibility. Exclusion criteria included being pregnant, having had diseases that may affect knee cartilage such as rheumatoid arthritis, or having a contraindication for MRI. There were 80 subjects excluded, and the remaining 449 subjects were asked to complete a short computer-assisted telephone interview with knee injury recorded in childhood and adulthood in response to the question “Have you had a knee injury requiring non-weight bearing treatment for more than 24 h or surgery?” They were requested to have an MRI scan at Epworth Hospital in Melbourne or North Shore Private Hospital in Sydney. Some participants (n = 119) did not undergo MRI after enrolling in the study due to the long distance that they would have needed to travel for MRI, work or family commitments, moving interstate, becoming pregnant by the time of MRI, or changing their mind. Two subjects’ MRI scans were not readable and they were excluded. This study was approved by the Southern Tasmania Health and Medical Human Research Ethics Committee, the Monash University Human Research Ethics Committee and the Northern Sydney and Central Coast Area Human Research Ethics Committee, and all participants provided written informed consent.

### Anthropometric measurements

Weight was measured to the nearest 0.1 kg in the CDAH study and in the CDAH Knee Cartilage study, with shoes, socks, and bulky clothing removed. Height was measured to the nearest 0.1 cm (with shoes and socks removed) using a stadiometer. Body mass index (BMI) was calculated as kilograms of weight per square metre of height.

### Physical activity measurements

Participants completed the long version of the International Physical Activity Questionnaire (IPAQ-L) in the CDAH study. Physical activity for leisure, transport, work and domestic purposes in the past week were assessed. Various domains of physical activities were considered including leisure and work-related physical activity. Physical activities were calculated to represent minutes per week of vigorous physical activity, moderate physical activity, walking and total physical activity. Regular participation is a key concept included in current public health guidelines for physical activity [19]. Therefore, both the total volume and the number of days/sessions are included in the IPAQ analysis algorithms. Three levels of physical activity, namely low, moderate, and high, are developed from the IPAQ-L according to the Guidelines for Data Processing and Analysis of the International Physical Activity Questionnaire [20]. The IPAQ demonstrates very good levels of repeatability and fair to moderate validity when compared to accelerometer data [21].

### Physical performance measures

PPMs, including long jump, leg muscle strength and physical work capacity at 170 heart beats (PWC_170_), were assessed in the CDAH study. Leg muscle strength was measured to the nearest 1.0 kg using dynamometers (TTM Muscle Meter, Tokyo, Japan). The dynamometer was adjusted to fit the size of each subject. Subjects were asked to stand on the leg dynamometer with a straight back, leaning on the wall and holding the bar hand with an overhand grip. Knees were flexed to 115°, at which the bar was attached to the dynamometer by a chain and the subject was asked to pull the chain by straightening the knee while keeping the back straight [22, 23].

The standing long jump was measured by asking the subject to stand on the gym mat with toes behind the line and with feet slightly apart. A two-feet take-off and landing was used, with the subject swinging the arms and bending the knees to provide the drive for the jump. The landing point at the closest part of the heel to the starting line was marked and the distance to the starting line was measured.

Cardiorespiratory fitness was estimated based on physical work capacity at a heart rate of 170 beats/minute, which was assessed using a Monark bicycle ergometer [24]. Subjects were asked to cycle at a constant 60 rpm for 3 min each at three successively increasing but submaximal workloads. Heart rate was recorded at one-minute intervals at each workload using an electronic heart rate monitor. PWC_170_ was assessed by linear regression with extrapolation of the line of best fit to a heart rate of 170 beats/minute. Repeatability was not assessed in our subjects but has previously been reported as having an intraclass correlation coefficient (ICC) of 0.92 [25]. A total of 261 subjects completed the VO_2max_ test and the correlation between PWC_170_ and VO_2max_ was 0.83 [22].

### MRI measurements

Participants had an MRI scan of their dominant knee after 5 years. MRI scans were obtained from two hospitals, which used the same type of machine (General Electric Medical Systems, Milwaukee, WI, USA). The knees were imaged in the sagittal plane on a 1.5-T whole body magnetic resonance unit with a commercial transmit-receive extremity coil. The following image sequence was used: a T1-weighted fat saturation, 3D spoiled gradient recall acquisition in the steady state; flip angle 55°; repetition time 58 msecs; echo time 12 msec; field of view 16 cm; 60 partitions; 512 × 512 matrix; acquisition time 11 min 56 s; one acquisition. Sagittal images were obtained at a partition thickness of 1.5 mm and an in-plane resolution of 0.31 × 0.31 (512 × 512 pixels).

Medial, lateral and total tibial cartilage volumes were determined by means of 3D image processing on an independent work station using the software program OsiriX (Geneva, Switzerland). Individual plates of tibial cartilage volume (medial and lateral) were isolated by manually drawing disarticulation contours around the cartilage boundaries on a section-by-section basis. These data were then re-sampled by means of bilinear and cubic interpolation (area of 312 × 312 μm^2^ and thickness of 1.5 mm, continuous sections) for the final 3D rendering. The coefficients of variation (CVs) for cartilage volume measures were 2.1–2.6 % [26]. Total tibial cartilage volume was calculated as the sum of the medial and lateral tibial cartilage volume.

The bone area of the medial and lateral tibial plateau was measured manually on the three reformatted T1-weighted MR images closest to the tibial cartilage in the axial plane as described in previous studies [27, 28]. The CVs for these measures were 2.2–2.6 % [26]. Total tibial bone area was calculated as the sum of the medial and lateral area.

Ten volunteers had MRI scans performed at both hospitals to determine the variation between machines. Bland–Altman plots were used to examine the observed difference between these machines and this difference was dependent on the magnitude of the reading. Based on these 10 volunteers, we calculated the correction equations for cartilage volume and bone area using the slope and intercept from a linear regression model, where the result from one machine was regressed on the result from the other machine.

### Statistical analyses

Mean and standard deviation or the percentages of the subjects were used for calculating the characteristics of the participants. Linear regression analyses were employed to examine the associations of physical activity and PPMs with adult tibial cartilage volume/bone area. Age at CDAH study, gender, duration of follow up in the CDAH Knee Cartilage study, BMI in the CDAH Knee Cartilage study, knee injury and/or tibial bone area were examined as potential confounders. Interactions between sex and predictor variables were examined in the regressions of tibial cartilage volume/bone area. All statistical analyses were performed using SPSS 19 for Mac (SPSS Inc., Chicago, IL, USA).

## Results

### Characteristics of the subjects

Information on the demographic and study factors of the 328 study participants is presented in Table 1. Male participants had higher total tibial cartilage volume, total tibial bone area and PPMs such as PWC_170_, leg muscle strength and long jump. Total physical activity, proportion of IPAQ high activity category, knee injury and vigorous physical activity were also significantly greater in male than in female participants. There were no significant differences in terms of age and BMI. There were no significant interactions between sex and predictor variables on tibial cartilage volume (data not shown) so we combined male and female participants for all analyses.

**Table 1:** Characteristics of the participants based on gender

	Female	Male	*P* value
	*n* = 155	*n* = 173	
Age, years	35.3 (2.7)	35.4 (2.6)	0.275
Tibial cartilage volume, cm^3^	3.2 (0.7)	4.5 (0.9)	<0.001
Duration of follow up, years	4.5 (1.2)	4.6 (1.2)	0.049
Body mass index, kg/m^2^	25.0 (4.6)	26.4 (3.7)	0.052
Tibial bone size, cm^2^	27.9 (2.4)	35.7 (3.3)	<0.001
Knee injury, %	11.3	22.9	0.005
5 years prior			
IPAQ category, high, %	34.5	49.0	0.008
Total physical activity, min/week	639.1 (403.1)	760.6 (566.3)	0.032
Vigorous physical activity, min/week	95.6 (169.1)	195.5 (240.8)	<0.001
Moderate physical activity, min/week	302.1 (275.6)	275.5 (241.7)	0.376
Walking, min/week	239.2 (215.2)	289.6 (299.6)	0.098
PWC_170_, per W	138.5 (30.1)	202.6 (46.5)	<0.001
Leg muscle strength, per kg	92.5 (26.6)	165.0 (34.3)	<0.001
Long jump, per cm	141.9 (25.9)	190.7 (24.0)	<0.001

Results are mean (SD) or percentage. The two-tailed *t* test was used to test for differences between means; the chi-square test was used for proportions (percentages). *IPAQ* international physical activity questionnaire, *PWC*_*170*_, physical work capacity at 170 beats per minute

### Physical activity and tibial cartilage volume and bone area

**Fig. 1:**
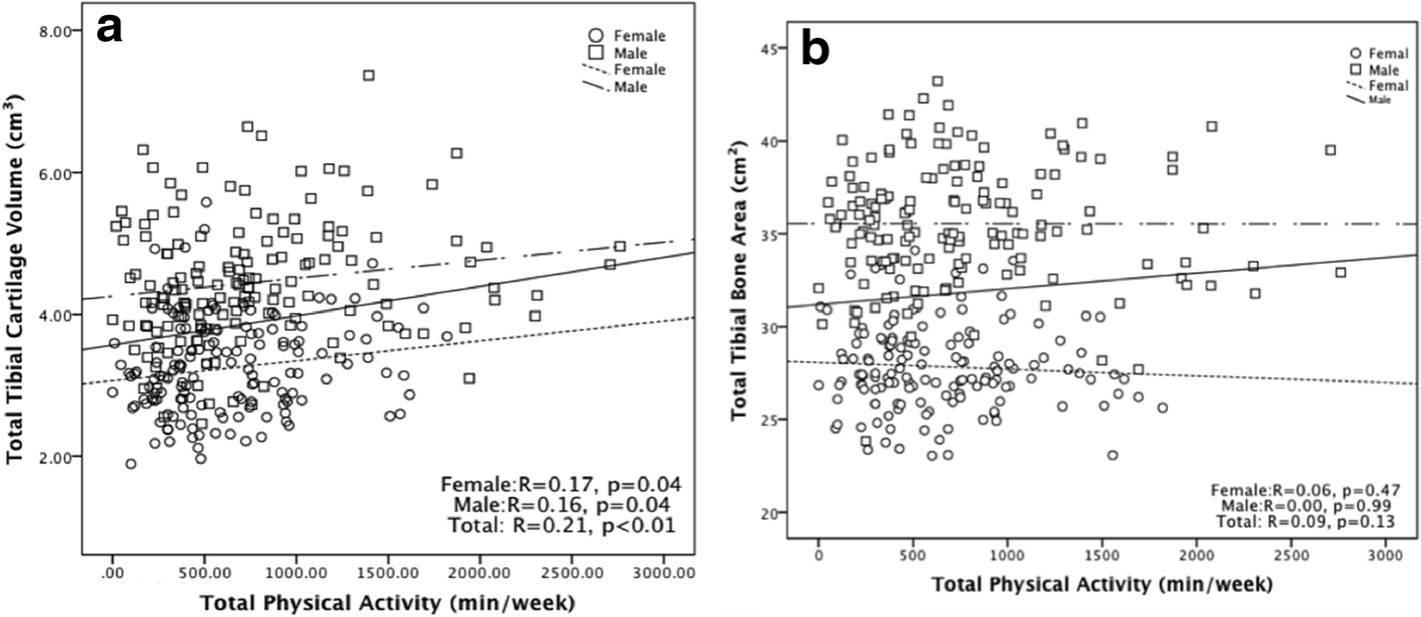
Association between total physical activity and total tibial cartilage volume (**a**) or tibial bone area (**b**)

Total physical activity measured using the IPAQ-L 5 years prior was positively associated with total tibial cartilage volume in univariable (Fig. 1a) and multivariable analysis (Table 2). Vigorous physical activity, walking and the IPAQ category (low, moderate, high) were positively associated with total tibial cartilage volume after adjustment for age, sex, BMI, injury, and duration of follow up, and remained significant after further adjustment for total tibial bone area (Table 2). The association between moderate physical activity and total tibial cartilage volume became significant after further adjustment for total tibial bone area (Table 2). Work-related physical activity measured 5 years prior was also beneficially associated with total tibial cartilage volume (*β* 0.41 mm^3^, 95 % CI 0.16, 0.66). These associations remained largely unchanged after further adjustment for the fitness measure PWC_170_. When, medial and lateral tibial cartilage volume was analyzed separately, the significant associations persisted largely for lateral tibial cartilage volume but not for medial tibial cartilage volume (Table 2).

**Table 2:** Association between physical activities measured 5 years prior and tibial cartilage volume

	Univariable	Multivariable^a^	Multivariable^b^
Cartilage volume (mm^3^)	*β* (95 % CI)	*β* (95 % CI)	*β* (95 % CI)
**IPAQ Category (high vs moderate and low)**			
Total tibial	**276.90 (103.42, 450.39)**	**195.83 (56.27,335.38)**	**182.94 (51.84,314.04)**
Medial tibial	**171.64 (40.28, 303.01)**	116.40 (−4.55, 237.36)	105.84 (−9.69, 221.38)
Lateral tibial	**300.61 (151.29, 449.93)**	**206.44 (77.93, 334.96)**	**198.74 (72.74, 324.75)**
**Total PA (min/week)**			
Total tibial	**0.41 (0.17, 0.63)**	**0.28 (0.10, 0.46)**	**0.30 (0.13, 0.47)**
Medial tibial	0.13 (0.00, 0.26)	0.07 (−0.05, 0.19)	0.08 (−0.03, 0.20)
Lateral tibial	**0.28 (0.13, 0.43)**	**0.21 (0.08, 0.33)**	**0.19 (0.07, 0.32)**
**Vigorous PA (min/week)**			
Total tibial	**1.18 (0.67, 1.69)**	**0.55 (0.12, 0.98)**	**0.54 (0.13, 0.94)**
Medial tibial	0.20 (−0.10, 0.51)	−0.08 (−0.35, 0.22)	−0.07 (−0.35, 0.20)
Lateral tibial	**0.98 (0.64, 1.31)**	**0.61 (0.31, 0.91)**	**0.55 (0.26, 0.84)**
**Moderate PA (min/week)**			
Total tibial	0.18 (−0.25, 0.62)	0.30 (−0.05, 0.65)	**0.34 (0.01, 0.67)**
Medial tibial	0.16 (−0.10, 0.41)	0.18 (−0.05, 0.41)	0.20 (−0.02,0.42)
Lateral tibial	0.03 (−0.26, 0.32)	0.12 (−0.13, 0.37)	0.11 (−0.14, 0.36)
**Walking PA (min/week)**			
Total tibial	**0.50 (0.07, 0.93)**	**0.35 (0.01, 0.069)**	**0.40 (0.07, 0.72)**
Medial tibial	0.18 (−0.07, 0.43)	0.12 (−0.10, 0.35)	0.15 (−0.06, 0.37)
Lateral tibial	**0.32 (0.04, 0.61)**	0.22 (−0.02, 0.47)	0.22 (−0.18, 0.46)

^a^Adjusted for age, sex, duration of follow-up, body mass index, knee injury. ^b^Further adjusted for corresponding tibial bone area. Bold denotes statistical significance at *p* <0.05. *IPAQ* International physical activity questionnaire, *PA* physical activity

Total physical activity assessed 5 years prior was not significantly associated with tibial bone area (Fig. 1b). None of these physical activity measures were significantly associated with tibial bone area in multivariable analysis after adjustment for age, sex, BMI, injury, and duration of follow-up (data not shown).

### Physical performance measures and tibial cartilage volume and bone area

**Fig. 2:**
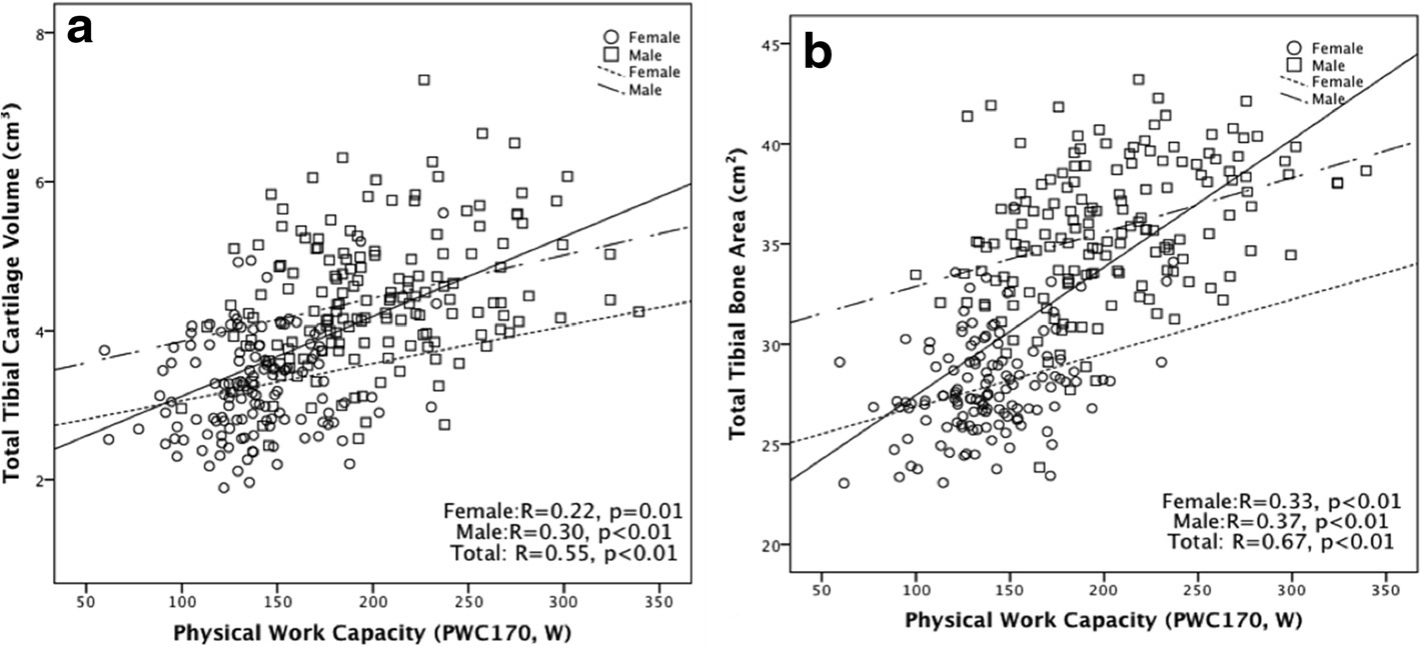
Association of physical work capacity at 170 heart beats (*PWC*_*170*_) with total tibial cartilage volume (**a**) and tibial bone area (**b**)

All PPMs were positively associated with total, medial and lateral tibial bone area in univariable analysis (Fig. 2b, Table 3). These associations remained significant after adjustment for age, sex, BMI, injury and duration of follow up except for the association between long jump and medial tibial bone area (Table 3). Adjusting for physical activity levels did not make any difference to these associations (data not shown).

**Table 3:** Association between physical performance measured 5 years prior and tibial bone area

Bone area, (mm^2^)	Univariable	Multivariable^a^
	*β* (95 % CI)	*β* (95 % CI)
**Long Jump (per cm)**		
Total tibial	**8.51 (7.20, 9.83)**	**1.94 (0.44, 3.45)**
Medial tibial	**4.19 (3.43, 4.94)**	0.71 (−0.19, 1.61)
Lateral tibial	**4.33 (3.56, 5.09)**	**1.23 (0.26, 2.21)**
**PWC**_**170**_ **(per W)**		
Total tibial	**6.39 (5.57, 7.21)**	**2.83 (2.00, 3.66)**
Medial tibial	**3.15 (2.67, 3.63)**	**1.37 (0.87, 1.87)**
Lateral tibial	**3.24 (2.37, 3.73)**	**1.47 (0.91, 2.02)**
**Leg muscle strength (per kg)**		
Total tibial	**7.18 (6.35, 8.01)**	**2.17 (1.07, 3.26)**
Medial tibial	**3.74 (3.27, 4.21)**	**1.36 (0.72, 2.00)**
Lateral tibial	**3.44 (2.92, 3.66)**	**0.81 (0.08, 1.54)**

^a^Adjusted for age, sex, duration of follow up, body mass index, knee injury. Bold denotes statistical significance at *p* <0.05. *PWC*_*170*_ physical work capacity at 170 heart beats

All PPMs including PWC_170_ (Fig. 2a) were positively associated with total tibial cartilage volume in univariable analysis (Table 4). These associations remained significant after adjustment for age, sex, BMI, knee injury and duration of follow up. The magnitudes of these associations decreased by 30–47 %, and the association of leg muscle strength became of borderline significance after further adjustment for total tibial bone area (Table 4). These associations remained unchanged after further adjustment for total physical activity (data not shown). When medial and lateral tibial cartilage volume were analyzed separately, the significant associations were more evident for lateral tibial cartilage volume (Table 4).

**Table 4:** Association between physical performance measured 5 years prior and tibial cartilage volume

	Univariable	Multivariable^a^	Multivariable^b^
Cartilage Volume (mm^3^)	*β* (95 % CI)	*β* (95 % CI)	*β* (95 % CI)
**Long Jump (per cm)**			
Total tibial	**14.85 (11.99, 17.70)**	**6.06 (2.14, 9.98)**	**4.26 (0.54, 7.97)**
Medial tibial	**6.36 (4.52, 8.19)**	2.51 (−0.15, 5.17)	1.42 (−1.13, 3.98)
Lateral tibial	**8.49 (6.49, 10.49)**	**3.55 (0.74, 6.36)**	**2.83 (0.04, 5.63)**
**PWC**_**170**_ **(per W)**			
Total tibial	**10.69 (8.83, 12.56)**	**5.71 (3.46, 7.96)**	**3.45 (1.13, 5.77)**
Medial tibial	**4.54 (3.32, 5.77)**	**2.56 (1.01, 4.11)**	1.04 (−0.57, 2.65)
Lateral tibial	**6.15 (4.85, 7.46)**	**3.15 (1.53, 4.78)**	**2.41 (0.67, 4.14)**
**Leg muscle strength (per kg)**			
Total tibial	**11.53 (9.55, 13.51)**	**4.16 (1.24, 7.09)**	2.20 (−0.63, 5.03)
Medial tibial	**5.35 (4.10, 6.61)**	**2.81 (0.86, 4.76)**	1.67 (−0.24, 3.58)
Lateral tibial	**6.18 (4.76, 7.60)**	1.35 (−0.75, 3.45)	0.53 (−1.59, 2.64)

^a^Adjusted for age, sex, duration of follow up, body mass index, knee injury. ^b^Further adjusted for bone area. Bold denotes statistical significance at *p* <0.05. *PWC*_*170*_ physical work capacity at 170 heart beats

## Discussion

To our knowledge, this is the first study that explored the associations between physical activity and physical performance, and knee cartilage volume and bone area in young adults aged 31–41 years. We found that the physical activity and PPMs were positively associated with total tibial cartilage volume. While PPMs were significantly associated with tibial bone area, there was no significant association between physical activity and tibial bone area.

Tibial cartilage volume is measured using MRI and it is associated with structural and functional characteristics of knee OA. Participants in our study were of mean age 36 years, and cartilage volume loss usually starts around the age of 40 years [29]. Therefore, these predominantly healthy subjects are expected to have reached their lifetime peak cartilage volume. Peak cartilage volume in the absence of other knee structural abnormalities predominantly reflects healthier cartilage. Similar to peak bone mass, which is a strong predictor of future risk of osteoporosis in older people [30], peak cartilage volume in a younger age group should be protective against the development of OA in later life. Furthermore, studies suggest that increased cartilage volume is associated with reduced radiographic evidence of OA [26, 31], cartilage defects [31] and knee symptoms [32].

Tibial bone area or cross-sectional tibial plateau surface area is a dynamic structure that may respond to physical stimuli [33]. Tibial bone area is positively associated with cartilage defects and cartilage volume loss in older adults [9], and has been shown to be an independent predictor of need for knee replacement over 4 years [34]. Increased tibial bone area in older populations appears maladaptive and is possibly due to disproportionate load transmission on the bone [8]. In contrast, exercise during childhood and adolescence is associated with increased cortical bone size which persists over years, leading to increased bone mass [7, 35]; therefore, increased bone area during growth may reflect adaptive change. Hypothetically, a greater tibial plateau will limit stresses in the joints because higher loads are distributed over a greater surface [36]. This is consistent with the findings from a cross-sectional study in which tibial bone area was found to be larger in athletes than in control subjects [8].

We have previously reported that physical activity in children with no injury is cross-sectionally and longitudinally associated with increased cartilage volume [3, 12]. The most striking association was with vigorous activity in the last two weeks: children undertaking any vigorous activity had 22–25 % greater cartilage volumes compared with those who did not undertake vigorous activity. Racunica et al. reported that tibial cartilage volume increased with the frequency and duration of vigorous activity reported 10 years previously, and with recent vigorous activity in the 7 days prior to MRI in healthy, community-based adults with no history of knee injury or disease [37]. Another study found that in the subgroup with no significant patella cartilage defects at baseline, participation in vigorous physical activity was associated with a reduced annual loss of patellar cartilage volume and a trend for fewer new patella cartilage defects [38]. Participation in exercise that causes tachypnea and an increased pulse rate for at least 20 min was associated with greater medial tibial cartilage volume in non-healthcare-seeking women at midlife [39]. In contrast, a cross-sectional study of young adult triathletes reported no difference in knee cartilage volume between triathletes and inactive volunteers, although knee bone area was generally larger in triathletes [8]. Another cross-sectional study in healthy older men found that physical activity was negatively associated with medial tibial cartilage volume [6]. Both these studies were small in sample size; however, in older subjects with abnormalities in the knee joint, such as bone marrow lesions or lower cartilage volume, persistent participation in vigorous physical activity was associated with adverse effects on cartilage in the medial compartment [38, 40, 41]. All these suggest that the effects of vigorous physical activity on knee structures may depend on age or the preexisting health of the joint.

The prevalence of structural abnormalities in this healthy young adult sample was very low (<15 %) and we found that all the physical activity measures including vigorous, moderate and total physical activity measured 5 years prior were positively associated with total tibial cartilage volume. This was largely independent of tibial bone area and fitness levels, suggesting that physical activity is beneficial to maintain healthy knee cartilage in younger adults. This can be explained in line with the studies that reported a positive effect of moderate exercise on glycosaminoglycan content in knee cartilage using delayed gadolinium enhanced MRI of cartilage (dGEMRIC) [42, 43]. However, our associations were mainly seen in the lateral tibial compartment and not in the medial tibial compartment. The reasons for this variation are unknown and could be due to a greater amount of cartilage in the lateral compartment [3] compared to the medial compartment, which may be more responsive to environmental stimuli such as physical activity. It was also noted that there was no significant association between any of the physical activity measures and tibial bone area, suggesting that tibial bone area may not be as responsive as tibial cartilage volume to physical stimuli in younger adults.

Muscle-strengthening intervention is now a major component of the usual treatment program for patients with knee OA, despite scant information about the effect on disease progression [44]. Studies report that increased muscle mass is associated with medial tibial cartilage volume and reduction in the loss of tibial cartilage [45], and physical fitness, especially PWC_170_, was associated with reduced loss of cartilage volume over time and was positively associated with change in tibial bone area at the lateral and total sites [16]. In the current study, we found that PWC_170_, leg muscle strength and long jump were positively associated with total tibial cartilage volume. Performance measures, especially PWC_170_, were more strongly correlated (*r* = 0.55) with cartilage volume than physical activity (*r* = 0.21). This could be partly due to the objective measurement of the PWC_170_ measure compared to subjective measurement of physical activity by questionnaire. Additionally, physical performance measures and cartilage volume may share genetic components while physical activity does not. This is evident from the finding that PWC_170_ was strongly correlated with tibial bone area (*r* = 0.67) and physical activity was not (*r* = 0.09). We also found that tibial bone area partly explained the association between PPMs and cartilage volume. These results were consistent with our previous findings in children where leg muscle strength was positively associated with cartilage volume [11] and tibial bone area [46]. We also found that childhood physical performance measures, especially PWC_170_, was positively associated with tibial bone area and cartilage volume in adults independent of adulthood fitness levels [46]. These associations between childhood PPMs and adult knee cartilage volume were also partially mediated by tibial bone area [46], suggesting that the higher PPMs are linked to greater knee cartilage volume, in part, through the development of knee bone area.

A strength of our study was the use of a population-based sample that is largely representative of young Australian adults. This study has several potential limitations. The response rate was low with only 43 % of the persons invited to participate having MRI performed. Reassuringly, there were no significant differences in age, sex, BMI, and knee injury between those with and without MRI scans, or between subjects included in this study and the remainder of the original cohort, which suggests that major selection bias was not introduced. We did not measure knee malalignment in this study and could not account for the influence of alignment on cartilage volume. Physical activity and performance measures were assessed 5 years prior, and we did not have MRI measurements at that time. Therefore, causality cannot be established. We have previously reported the loss of cartilage volume starts at the age of 40 years. Therefore, it is expected that our subjects may have a similar cartilage volume 5 years prior.

## Conclusions

In conclusion, while total tibial bone area is affected only by physical performance, total tibial cartilage volume can be influenced by both physical activity and physical performance in younger adults. The clinical significance suggests a beneficial effect of physical activity for cartilage but the bone area association was restricted to physical performance suggesting other factors rather than physical activity may be important.

## Abbreviations

BMI: body mass index
CDAH: childhood determinants of adult health
CV: coefficient of variation
dGEMRIC: delayed gadolinium enhanced magnetic resonance imaging of cartilage
ICC: intraclass correlation coefficient
IPAQ: International physical activity questionnaire
MRI: magnetic resonance imaging
OA: osteoarthritis
PPMs: physical performance measures
PWC_170_: physical work capacity at 170 heart beats
